# What is the best path towards allogeneic transplantation in MDS and AML? A survey among German-spreaking centers for allogeneic hematopoietic stem cell transplantation

**DOI:** 10.1007/s00277-026-07115-9

**Published:** 2026-06-06

**Authors:** Stefan W. Krause, Wolfgang Bethge, Gesine Bug, Ahmet Elmaagacli, Edgar Jost, Stefan A. Klein, Guido Kobbe, Sabrina Kraus, William H. Krüger, Thomas Luft, Lutz P. Müller, Kerstin Schäfer-Eckart, Johannes Schetelig, Normann Steiner, Matthias Stelljes, Johanna Tischer, Julia Winkler, Daniel Wolff, Friederike Wortmann, Christoph Röllig

**Affiliations:** 1 Department of Medicine 5, Uniklinikum Erlangen, Erlangen, Germany; 2 Department of Hematology, Oncology, Clinical Immunology and Rheumatology, University Hospital Tübingen, Tübingen, Germany; 3 Department of Medicine 2, Goethe University Frankfurt, University Hospital, Frankfurt am Main, Germany; 4 Abteilung für Hämatologie/Onkologie und Stammzelltransplantation, Asklepios Klinik St.Georg, Hamburg, Germany; 5 Department of Hematology, Oncology, Hemostaseology and Stem Cell Transplantation, Faculty of Medicine, RWTH Aachen University, Aachen, Germany; 6 Department of Haematology and Oncology, University Hospital Mannheim, Heidelberg University, Mannheim, Germany; 7 Department of Hematology, Oncology and Clinical Immunology, University Hospital Düsseldorf, Medical Faculty, Heinrich Heine University, Düsseldorf, Germany; 8 Department of Internal Medicine II, University Hospital of Wuerzburg, Wuerzburg, Germany; 9 Klinik für Innere Medizin C, Universitätsmedizin Greifswald, Greifswald, Germany; 10 Department of Hematology and Oncology, University of Heidelberg, Heidelberg, Germany; 11 Universitätsklinik für Innere Medizin IV, Universitätsklinikum Halle, Halle, Germany; 12 Department of Internal Medicine 5, Klinikum Nuernberg, Paracelsus Medizinische Privatuniversität, Nürnberg, Germany; 13 Medizinische Klinik und Poliklinik I, Universitätsklinikum TU Dresden, Dresden, Germany; 14 Department of Internal Medicine V, Medical University of Innsbruck, Innsbruck, Austria; 15 Department of Medicine A, Hematology, Oncology, and Pneumology, University Hospital Münster, Münster, Germany; 16 Department of Internal Medicine III, LMU, University Hospital Munich, Campus Grosshadern, Munich, Germany; 17 Department of Internal Medicine III, University Hospital Regensburg, Regensburg, Germany; 18 Klinik für Hämatologie und Onkologie, UKSH Campus Lübeck, Lübeck, Germany

**Keywords:** Hematopoietic stem cell transplantation, Standard of care, Bridging, Induction chemotherapy

## Abstract

Allogeneic hematopoetic stem cell transplantation (allo SCT) is a treatment option with a unique chance of cure for patients with high-risk AML or MDS. However, the optimal path for an individual patient on its way to allo SCT is far from clear and subject of ongoing debates. Upfront transplantation “as soon as possible” competes with strategies to achieve deep remission or at least stabilization of the disease. In this context, we performed a survey among German transplant centers to assess their preferred strategies for patients before SCT in five typical scenarios of AML and MDS. We obtained replies from 22 centers, revealing a heterogenous use of possible strategies. Upfront transplantation was a preferred option (68%) in intermediate risk MDS, chemotherapy was preferred (64%) as bridging for AML in remission, whereas hypomethylating agents (HMA) and induction chemotherapy for relapsed AML were highly controversial. Further prospective evaluation of different options is desirable.

**Supplementary Information:**

The online version contains supplementary material available at 10.1007/s00277-026-07115-9.

## Introduction

For many patients with high-risk AML or MDS, allogeneic hematopoetic stem cell transplantation (allo SCT) is a curative treatment option of crucial importance. Indications for allo SCT depending on the trade-off of individual disease-specific and procedure-related risks have been discussed extensively in the literature and are not the topic of this report. However, once the indication for allo SCT has been established for an individual patient, the question arises whether the transplantation should be performed “upfront” or whether some “induction” or “bridging” therapy should be applied first. In AML, the achievement of disease remission prior to allo SCT is associated with favorable long-term survival for patients actually undergoing transplantation (e.g [1, 2]). However, attempts to achieve remission before allo SCT come at a price: acute toxicity of intensive chemotherapy and infections which may lead to complications that ultimately preclude transplantation, use of antibiotics may lead to shifts in the microbiome and inferior transplant outcomes [3], and the risk to select more resistant neoplastic clones may increase relapse rates or render relapses refractory to common salvage strategies [4, 5]. Thus, the optimal timing of transplantation is unclear, as is the optimal strategy in the event of induction therapy failure or in AML recurrence [6]. In MDS, a stable disease without blast excess indicates a more favorable prognosis in transplanted patients [7], but it is unclear whether (and if so, how) such a situation should be attempted using upstream therapies for bridging or if it may be preferrable to perform upfront transplantation without bridging and avoid dropout of patients on their way to transplantation [8–11]. Azacitidine is an important therapeutic option for relapsed disease after allo-SCT, either alone or in combination with donor lymphocyte infusions [12]. This therapeutic approach may be less effective, if HMA treatment has been used pre-transplant [5, 11, 13]. In this situation, we aimed to gather information on strategies currently preferred in German-speaking transplant centers on the patients’ path to allo SCT.

## Methods

In a survey among transplant centers caring for adult patients, the usual procedures were inquired for patients with AML and high-risk MDS for whom an indication for allo SCT had been established. The survey asked which therapy was administered in an individual patient between the decision to perform allo SCT and the start of conditioning therapy for transplantation in several different scenarios. SWK created an initial version of the questionnaire, which was modified and finalized following feedback from several other centers. The paper-based questionnaire was sent to the allogeneic transplant centers of Study Alliance Leukemia (SAL) and the German Working Group of Hematopoetic Stem Cell transplantation and Cellular Therapies (DAG-HSZT) in 2021. Each transplant center was asked to provide one single response per center.

The following prerequisites were assumed for all scenarios described in the questionnaire: the indication for allo SCT had already been agreed upon in the transplant center, taking into account the individual patient’s age, general condition and comorbidities. Transplantation was assumed to be feasible without restrictions (not borderline due to age or general condition), the patient agrees to the transplant after informed consent and there are good chances of finding a suitable fully matched donor.

Participants were asked to rate on a 4-point Likert scale („yes - vast majority of cases“ / „majority of cases“ / „occasionally“ / „no - never or very rarely“) whether several possible bridging options between indication to perform an allo SCT and start of conditioning treatment were used in their centers. Five different scenarios were described, together with 3–6 corresponding options for bridging to transplant (Table 1). In addition, participants had the opportunity to provide reasons for their preferred options. At the time of the survey, the “ASAP” trial [6], which randomly tested intensive chemotherapy for AML relapses before transplantation, was still open for recruitment, as was the PALOMA study, which tested the use of CPX-351 in high-risk MDS and AML with low blast counts within a randomized trial. A follow-up survey was sent out to the respondents in March 2026, containing the same questions with slight modifications: questions regarding ASAP and PALOMA trials (that were closed in the meantime) were deleted and a question on HMA plus venetoclax in refractory AML was introduced.

The replies were transferred to an Excel spreadsheet for descriptive statistics. “yes” and “majority of cases” together are described as “preferred option” in the manuscript. Answers that could not be interpreted unambiguously were clarified by personal consultation. The graphical display of the reply frequencies was done using GraphPad Prism.

**Table 1:** Treatment options in 5 different scenarios to be rated in the survey

Scenario 1, MDS EB-1. In the patient, an MDS is newly diagnosed. Cytologically, a blast excess is found, just below 10%. Together with the other risk factors, an IPSS-*R* of 4 points (intermediate risk) is calculated.
• We try to identify a suitable donor quickly and transplant the patient “upfront” without any specific therapy in between.
• We initiate a therapy with hypomethylating agents (HMA) in order to halt the progression of the disease as far as possible. This therapy strategy only serves as a bridging measure, preferably only 1–2 cycles.
• We initiate a therapy with HMA to halt disease progression or, ideally, to achieve remission and, if successful, to postpone the transplant. The final decision for transplantation is only made in the event of deterioration or lack of improvement.
Scenario 2, MDS EB-2. In a 60-year-old patient with newly diagnosed MDS, a blast proliferation of just below 15% is found cytologically. Together with the other risk factors, an IPSS-R of 5.5 points (high risk) is calculated.
• We try to identify a suitable donor quickly and transplant the patient “upfront” without any specific therapy in between.
• We initiate a therapy with hypomethylating agents (HMA) in order to halt the progression of the disease as far as possible. This therapy strategy only serves as a bridging measure, preferably only 1–2 cycles.
• We initiate a specific therapy with hypomethylating substances to ideally achieve remission and then transplant.
• We carry out AML induction therapy to ideally achieve a remission and then transplant.
Scenario 3, AML with low blast count. In a 60-year-old patient, a history of MDS is documented. For the MDS only supportive therapy was applied so far. Cytology now shows a clear blast proliferation of just over 20%. The ELN 2017 risk is adverse with cytogenetics showing an isolated loss of chromosome 7.
• We try to identify a suitable donor quickly and transplant the patient “upfront” without any specific therapy in between.
• We initiate a therapy HMA in order to halt the progression of the disease as far as possible. This therapy strategy only serves as a bridging measure, preferably only 1–2 cycles.
• We initiate a therapy with HMA plus Venetoclax to ideally achieve remission and then transplant.
• We perform AML induction therapy with CPX-351 (Vyxeos) to ideally achieve remission and then transplant.
• We perform AML induction therapy with Daunorubicin plus AraC or variants thereof to ideally achieve remission and then transplant.
• We include the patient in the PALOMA trial (comparison of CPX-351 based treatment versus conventional care).
Scenario 4, AML in remission. A 65-year-old patient with newly diagnosed AML has received one cycle of induction therapy. The ELN 2017 risk is adverse with an isolated − 7. Cytologically, a CR was achieved. A sibling donor is not available, but the search for an unrelated donor is promising according to a database search. Conditioning for SCT could realistically begin in 4 weeks.
• We aim to transplant the patient without any further therapy in between.
• We give one cycle of chemotherapy, e.g. HD-AraC, as consolidation before transplantation.
• We initiate a therapy with HMA in order to ideally halt progression before transplantation.
Scenario 5, refractory AML. A 65-year-old patient with newly diagnosed AML has received induction therapy. The ELN risk score is intermediate. Cytology shows a significant reduction in the number of blasts in the control bone marrow aspirate, but these are still above 5%. In addition, flow cytometry and/or molecular biology confirm the persistence of AML. A donor is identified. The start of conditioning for SCT could realistically begin in 3 weeks.
• We aim to transplant the patient without any further therapy in between, potentially using sequential conditioning for transplantation. If disease progression should occur in the meantime, we give a mild cytoreductive therapy (e.g. hydroxyurea or AraC) as bridging.
• We initiate a therapy with HMA in order to ideally halt progression before transplantation.
• We give one cycle of chemotherapy, e.g. HD-AraC, as consolidation before transplantation.
• We give one cycle of 2nd line chemotherapy with High-dose AraC - mitroxantrone, FLAG-Ida or similar before transplantation.
• We suggest the ASAP trial (immediate transplantation vs. 2nd line therapy for remission induction) to the patient.

## Results and discussion

Twenty-two centers (one from Austria) including several high-volume institutions responded to our survey, covering close to half (46% in 2022) of the allo SCTs performed in Germany. Many questionnaires were explicitly completed jointly by several physicians of the transplant teams. After personal consultation with some participants concerning a few ambiguous responses, all replies in all questionnaires were evaluable. A cross-check of the replies confirmed overall plausibility, i.e. if respondents rated an option as preferred, the opposite strategy was mostly rated as rarely or never used.

**Fig. 1:**
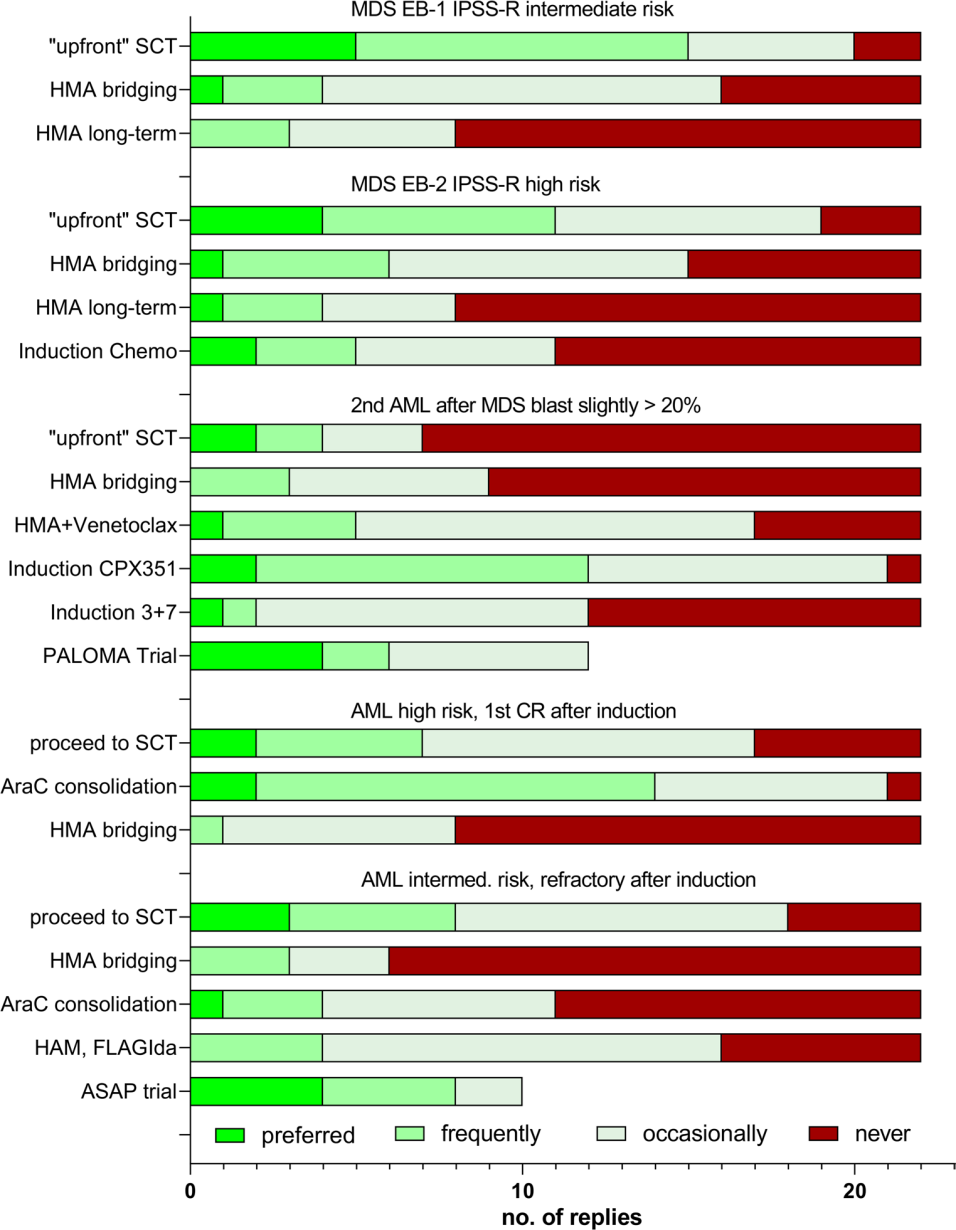
Treatment options for patients with MDS or AML on their path towards allogeneic SCT in 5 different scenarios as rated by members of 22 transplant centers. Reply "never" regarding the PALOMA or ASAP trials is not displayed, because the respective trial may be just unavailable at a center

Results for all scenarios are depicted in Fig. 1. The participants’ responses were extremely heterogeneous. In all scenarios and for almost any of the proposed options, some participants indicated that they would never use that option and others considered the same option to be their preferred strategy.

Regarding MDS, upfront transplantation was the preferred strategy (68% in intermediate risk, 50% in a high risk scenario) in many centers but nevertheless rated as never used by a minority of participants. Longer term HMA was not acceptable to the majority (64%) of participants, but preferred by a relevant minority (14% and 18%), whereas attitudes toward short term HMA were mixed. Induction chemotherapy for MDS EB-2 was never used by 50% of the participants but occasionally used or even a preferred option for the other half of the centers. These results are in line with published data. Whereas HMA improved the prognosis of patients with higher risk MDS not suitable for intensive therapy [14], its use may even be disadvantageous before SCT [11, 13].

In secondary AML with a history of MDS and low blast counts, upfront transplantation was preferred (18%) or considered (14%) by a significant minority of centers, whereas induction with either CPX-351 (55%) or conventional Daunorubicin-Cytarabine (9%) was preferred by a majority. At the time of the survey, the PALOMA trial testing CPX-351 in a randomized phase II trial was recruiting. Results are expected for 2026. Interestingly, HMA plus Venetoclax was also rated as an occasionally used (55%) or even preferred (22%) option to be considered for patients scheduled for allo SCT in this scenario, although data from clinical trials [15] and licensing in Europe are referring to patients not suitable for intensive chemotherapy.

For patients with AML in remission after induction chemotherapy, the most commonly chosen approach (64%) for the waiting period until transplantation was bridging with a cycle of consolidation chemotherapy. Refraining from additional therapy before allo SCT was also frequently chosen (32%) but was not an option for a minority of centers. Bridging with HMA would only be used rarely, and not at all in the majority (64%) of centers.

For AML not in remission after induction, preferred strategies were diverse. The most prominent options, high-dose Cytarabine-based salvage chemotherapy or rapid initiation of transplantation were considered possible options by a large proportion of centers but both options were completely rejected by others. At the time of our survey, the ASAP study was still recruiting participants. In this randomized trial, in one treatment arm 2nd line re-induction treatment (high dose AraC plus Mitoxantrone) was omitted and upfront transplantation was performed using a “sequential” conditioning. Sequential conditioning was also used in the other treatment arm, if remission was not achieved with induction treatment. Results have been published in the meantime and showed that disease biology is the major predictor of success and 2nd line intensive chemotherapy did not lead to improved long-term outcomes in patients with relapsed or refractory AML [6]. However, new options have since emerged and were not included in our survey. The combination of Venetoclax and HMA, which has been successfully established in first-line therapy [15], has now been used in some institutions in second line as a bridge to transplant and has shown promising results, although these have so far only been shown in retrospective analyses [16]. Combinations of intensive chemotherapy and Venetoclax have also been reported with encouraging results [17, 18].

To determine whether the preferences of the centers had changed in the meantime, we asked the participants to answer the questions again. Nineteen participants complied with our request. There were only minor shifts in the responses compared to the first round of participation (suppl. Figure 1). Regarding MDS, there was a further slight increase in the rejection of longer-term therapy with HMA. For refractory AML, in line with data from the ASAP study, support for upfront transplantation rose from 42% to 64%, while intensive relapse chemotherapy was rejected more frequently (response “never”: 2021 32%, now 45%). HMA plus venetoclax was reported to be used only occasionally by part of the centers.

Respondents’ rating of reasons for potentially choosing immediate allo SCT vs. other options for individual patients were less informative. Donor availability, progression, patients’ preferences and molecular characteristics were rated as important decision factors (suppl. Figure 2). Some free text comments showed that the processes of decision making are more difficult to capture quantitatively in a survey than the decisions themselves.

Taken together, the responses show (albeit not unanimously) accepted preferences in various indications such as upfront transplantation in intermediate risk MDS, while in other common clinical constellations, there is great uncertainty and heterogeneity of approaches. The main reason for the latter phenomenon is that current evidence on the optimal path towards allo SCT for patients with myeloid neoplasms is still scarce, leading to highly heterogeneous treatment preferences in different centers. Regarding refractory and relapsed AML, data were provided recently by the randomized ASAP trial [6] and led to increased preference of direct transplantation vs. 2nd line intensive chemotherapy among our repondents, however, new therapeutic options are emerging for this situation and no data from randomized comparisons are available for other scenarios. Thus, further prospective trials are highly desirable.

## Supplementary Information

Below is the link to the electronic supplementary material.

Supplementary Material 1

## Data Availability

The original survey form in German as well as the modified survey form for the 2026 update are available upon request. The responses from the individual centers are confidential and cannot be made available without consulting the centers.
