# Supplementary material for: What is the best path towards allogeneic transplantation in MDS and AML? A survey among German-spreaking centers for allogeneic hematopoietic stem cell transplantation
Source: Ann Hematol. 2026 Jun 6;105(7):298. doi: 10.1007/s00277-026-07115-9 (PMC13241428; doi:10.1007/s00277-026-07115-9)
Supplement: Supplementary file 1 — Supplementary Material 1 [file 277_2026_7115_MOESM1_ESM.docx]

What is the best path towards allogeneic transplantation in MDS and AML?

A survey among German-spreaking centers for allogeneic hematopoietic stem cell transplantation

Brief report to Annals of Hematology

Stefan W. Krause^1^, Wolfgang Bethge^2^, Gesine Bug^3^, Ahmet Elmaagacli^4^, Edgar Jost^5^, Stefan A. Klein^6^, Guido Kobbe^7^, Sabrina Kraus^8^, William H. Krüger^9^, Thomas Luft^10^, Lutz P. Müller^11^, Kerstin Schäfer-Eckart^12^, Johannes Schetelig^13^, Normann Steiner^14^, Matthias Stelljes^15^, Johanna Tischer^16^, Julia Winkler^1^, Daniel Wolff^17^, Friederike Wortmann^18^, Christoph Röllig^13^

# Supplementary figures:

**Suppl. fig. 1.** Follow up results from spring 2026 on preferences regarding treatment options for patients with MDS or AML on their path towards allogeneic SCT in 5 different scenarios as rated by members of 19 transplant centers. HMA+Venetroclax is now included as an option in refractory AML.

**suppl Fig 2** relevance of reasons for considering "upfront" allo SCT in a specific scenario.
